# Supplementary material for: Comparative multiomics analysis of cell physiological state after culture in a basket bioreactor
Source: Sci Rep. 2022 Nov 23;12:20161. doi: 10.1038/s41598-022-24687-4 (PMC9686226; doi:10.1038/s41598-022-24687-4)
Supplement: Supplementary file 1 — Supplementary Information 1. [file 41598_2022_24687_MOESM1_ESM.zip › raw data/Transcriptomic raw data/6.Enrichment/2.KEGG/TE1vsTC1/ALL/src/csab04979.html]

csab04979


- csab:103234364

- Up regulated genes

103234364(1.37865418464166)

- csab:103234364

- Up regulated genes

103234364(1.37865418464166)

- csab:103234364

- Up regulated genes

103234364(1.37865418464166)

- csab:103234364

- Up regulated genes

103234364(1.37865418464166)

- csab:103234364

- Up regulated genes

103234364(1.37865418464166)

- csab:103229330

- Up regulated genes

103229330(1.0933059103651)

Close
